# A qualitative exploration of post-primary educators’ attitudes regarding the promotion of student wellbeing

**DOI:** 10.1080/17482631.2021.1946928

**Published:** 2021-06-30

**Authors:** David Byrne, Dr Aiden Carthy

**Affiliations:** Humanities, Technological University Dublin – Blanchardstown Campus, Dublin, Ireland

**Keywords:** Wellbeing, attitude, teacher, educator, school, thematic analysis, qualitative

## Abstract

**Purpose**: The success of measures aimed at promoting student health and wellbeing is greatly informed by the attitudes and perceptions of the educators assigned to implement these measures. The aim of this study was to examine the attitudes of post-primary educators in Ireland regarding the promotion of student wellbeing.

**Method**: Semi-structured interviews (n = 11) were conducted with post-primary educators located across the Republic of Ireland. Data was analysed using reflexive thematic analysis.

**Results**: Analysis resulted in the interpretation of five themes related to best practice, the value of wellbeing promotion, work-related time constraints, an atheoretical approach to wellbeing promotion and educators’ accounts of their own wellbeing. Participants shared numerous concerns regarding their ability to attend to student wellbeing, principal among which were a lack of appropriate training, work-related time constraints and a potential systematic de-valuing of the wellbeing curriculum relative to the core curriculum.

**Conclusion**: It is recommended that appropriate accreditation be necessary in order to register to teach any aspect of the wellbeing curriculum, and that a requisite wellbeing-orientated knowledge and skillset be mandatory for all educators in order to register as a post-primary teacher with the teaching council in Ireland.

## Introduction

In 2015, wellbeing was formally recognized as an area of learning in post-primary schools in the Republic of Ireland (Department of Education and Skills, 2015). A wellbeing curriculum has since been developed, which consists of Social, Personal and Health Education (SPHE), Civic, Social and Political Education (CSPE) and Physical Education (PE). Schools are afforded a high degree of autonomy in developing and implementing appropriate wellbeing policies, delivering relevant wellbeing curricula, and realizing appropriate whole-school wellbeing practices. To facilitate schools in this endeavour, the National Council for Curriculum and Assessment (NCCA) in Ireland released the NCCA wellbeing guidelines in 2017. Among other things, these guidelines; delineate wellbeing as a whole-school endeavour; discuss the different aspects of the school context that can influence student wellbeing, and; identify a number of “indicators of wellbeing” that can be used to monitor and report on student wellbeing outcomes. The aim of these guidelines is to “support schools in planning and developing a coherent wellbeing program that builds on the understandings, practices and curricula for wellbeing already existing in schools” (NCCA, 2017, p. 8). Positive educator attitudes and perceptions have been found to be among the most influential factors in achieving successful whole-school implementation of health- and wellbeing-orientated practices (Byrne et al., 2018; Mayock et al., 2007; NCCA, 2017). Indeed, the NCCA highlight the centrality of the educator in this regard by identifying that “wellbeing starts with the staff” (NCCA, 2017, p. 29). Numerous international studies contribute to this narrative and attribute significant importance to the meaning and meaningfulness educators ascribe to health and wellbeing practices.

One line of research, which was conducted in Cyprus, noted positive perceptions among educators with regard to the prospect of delivering health education. It was also observed that when teachers were positive about their own health, they tended to hold more favourable perceptions regarding the inclusion of health education in their schools’ curriculum (Fontana & Apostolidou, 2002). However, subsequent research by these authors found that teachers’ attitudes regarding health education were not wholly positive. The follow-up study did note the previously identified underlying positivity among educators, with 87% of educators found to be welcoming of the opportunity to deliver health education. However, 61% of educators also argued that their schools’ curriculum was too full to accommodate health education. Teachers also reported receiving inadequate training to deliver health education, with 82% arguing they needed more continuous professional development (CPD) in “health matters” and 84% citing a need for CPD in “the methodology of health education” (Apostolidou & Fontana, 2003). The authors of these studies subsequently recommended that teachers receive additional health education training, arguing this would foster positive attitudes among teachers regarding health education.

Teachers can also be uncomfortable with delivering aspects of the wellbeing curriculum. One study in Australia identified significant levels of discomfort among teachers who were tasked with delivering sexuality education. Some teachers were found to be particularly unforthcoming with regard to Lesbian, Gay, Bisexual, Transgendered, Queer, Inter-sex (LGBTQI) students due to a lack of training and a perceived ambiguity with regard to relevant school policy (Shannon & Smith 2015). However, teacher discomfort is not typically predicated upon the sensitivity of the subject being taught. In America, 50% of educators surveyed in one study indicated being uncomfortable and lacking confidence with regard to attending to students’ mental health in their classroom (Walter et al., 2006). Teachers in England expressed concerns regarding the changing nature of their responsibilities in school. Particular trepidation was noted regarding the requirement to attend to students’ emotional and psychological wellbeing and how this might affect teachers’ own emotional and psychological wellbeing (Rothì et al., 2008).

A trend of discomfort has also been observed among Irish educators with regard to the delivery of the wellbeing curriculum. Mayock et al. (2007) noted that, in 187 participating schools, teacher discomfort with delivering Relationship and Sexuality Education (RSE) was reported to be the most significant barrier to the appropriate implementation of this aspect of the wellbeing curriculum in 71% of schools. The school curriculum was also said to be too overcrowded to accommodate RSE by 82% of schools, with approximately two-thirds of schools further stating that delivering RSE added to the perceived pressure of delivering core curricular subjects. Perhaps unsurprisingly, aspects of the wellbeing curriculum, such as SPHE and its subsidiary components (e.g., RSE), are very often de-valued by educators in comparison to the core curriculum (Doyle, 2017; Mayock et al., 2007; O’Higgins et al., 2013).

In keeping with the international literature, Irish educators have been found to be somewhat aggrieved by the requirement to allocate time to wellbeing curricular activities that would have otherwise been spent on core curricular activities (O’Higgins et al., 2013). While Irish educators tend to embrace pastoral care as a fundamental responsibility for all school staff (Hearne & Galvin, 2014), the requirement to attend to student wellbeing in a formalized manner appears to be a cause of some discomfort, stress and resentment. This is further complicated when considering the difficulties in achieving appropriate levels of training among relevant staff, with as many as one third of SPHE teachers lacking any kind of formal training in this subject (Moynihan et al., 2016). There have been many arguments made that an appropriate level of training would not only improve the delivery of the wellbeing curriculum, but also improve educators’ perceptions of the wellbeing curriculum (see Doyle, 2017; Moynihan et al., 2016; O’Higgins et al., 2013). However, the lack of value ascribed to the wellbeing curriculum relative to the core curriculum, as well as time constraints related to an overloaded curriculum, have resulted in a reluctance among educators to pursue training related to the wellbeing curriculum (O’Higgins et al., 2013). In this regard, there would appear to be a self-fulfilling pattern of a lack of appropriate training resulting in a lack of value for the wellbeing curriculum, and a lack of value precipitating a reduced uptake in relevant training opportunities.

While there is an abundance of literature examining the implications of social and emotional learning (SEL) programmes and health and wellbeing curricula regarding wellbeing outcomes for students, there remains a relative dearth of research examining the attitudes and perspectives of the educators tasked with delivering these programmes and curricula. This is particularly true in the Irish context. While several studies have been conducted along these lines, they have tended to focus on specific cohorts or specific aspects of the wellbeing curricula (see Doyle, 2017; Hearne & Galvin, 2014; Mayock et al., 2007; Moynihan et al., 2016). The aim of this study was to conduct a holistic examination of the attitudes and perceptions of Irish post-primary educators with regard to the promotion of students’ social and emotional wellbeing. To the authors’ knowledge, this was also the first study of its kind since the introduction of the NCCA wellbeing guidelines.

## Method

The data presented in this paper represent the qualitative phase of a larger, mixed-methods study. The aims of this study were to: examine post-primary educators’ attitudes regarding the promotion of student wellbeing, as well as the NCCA wellbeing guidelines; identify potential barriers to best practice in the development of student wellbeing, and; identify changes that might augment the wellbeing curriculum. In this study, wellbeing was conceptualized by observing eudaimonic traditions of wellbeing, with particular emphasis upon the student/teacher dialectical framework (Deci & Ryan, 2000). Ethical approval for this study was granted by the ethics committee at Technological University Dublin—Blanchardstown Campus.

## Participants

An opportunistic sampling method was adopted for this study. A point of contact (typically a secretary or administrative assistant) at 724 Irish schools was emailed and informed of the upcoming interviews. Points of contact were requested to forward the email to their respective faculty so that any members of staff who may wish to participate in this phase could register their interest in being interviewed. In total, 11 educators were interviewed. A reasonably diverse representation was achieved in terms of gender, urban/rural status, subjects taught, and experience with wellbeing practices. However, teachers were over-represented, with only one vice-principal bucking the trend of positions reported. In addition, no respondents were situated within all-boys schools (see Table I). The remote nature of the location of two participants (P2 and P3) was such that they could not be interviewed face-to-face. Participant two was interviewed via Skype and participant three’s interview was conducted by phone. The use of online face-time software such as Skype has been demonstrated to be an appropriate analogue to conducting in-person interviews (Lo Iacono et al., 2016). It has also been demonstrated that telephone interviews are a viable option for collecting rich qualitative data. However, this option is limited by the absences of opportunity to record non-verbal communication (i.e. body language) (Drabble et al., 2015). At the conclusion of data collection, it was considered that saturation point had been achieved.

**Table I.** Participants

			All-boys/All-girls/Co.Ed.			
Participant	Gender	Urban/Rural		Position	WB Position	Subject(s) Taught
P1	Male	Rural	Co.Ed.	Teacher	-	Business studies and P.E.
P2	Male	Rural	Co.Ed.	Teacher	Prev. Temp. SPHE	Geography and Maths
P3	Female	Rural	Co.Ed.	V. Principal	Pastoral Care Team	None
P4	Female	Rural	Co.Ed.	Teacher	Wellbeing Coordinator	SPHE
P5	Female	Rural	Co.Ed.	Teacher	Prev. Temp. SPHE	English and History
P6	Female	Rural	Co.Ed.	Teacher	-	Geography and History
P7	Male	Urban	Co.Ed.	Teacher	-	English and Classical Studies
P8	Female	Rural	All-girls	Teacher	-	Maths and Science
P9	Female	Urban	All-girls	Teacher	Pastoral Care Team	SPHE and History
P10	Male	Urban	Co.Ed.	Teacher	-	CSPE and English
P11	Female	Rural	All-girls	Teacher	Pastoral Care Team	SPHE and Art
P12	Female	N/A	N/A	Teacher	N/A	N/A

## Procedure

Data was collected via semi-structured interviews. All participants were informed that interviews would be audio recorded and briefed of their right to withdraw from participation at any point and for any reason. Informed consent was obtained subsequent to completion of each interview. Interviews were largely inductive, with an emphasis upon emergent information, and guided by what the participant found to be meaningful regarding a particular topic. To remain “on topic”, an interview agenda (see Appendix A) was loosely adhered to, which addressed four distinct areas: the general task of promoting student wellbeing; the current wellbeing curriculum; the NCCA wellbeing guidelines, and; educators’ perceptions of their own wellbeing. The interview agenda was used as a reflexive tool to help ensure that while discourse remained subjective to the participant, it also remained relevant to the research questions. With consideration that some interviews might be arranged during “free periods” (which turned out to be the case for some participants), the interview agenda was designed to be completed in no more than 40 minutes, which is the typical duration of a class in post-primary education.

## Analysis

Reflexive thematic analysis (Braun & Clarke, 2014, 2019, 2020) was used to analyse data. Epistemological considerations for this analysis were constructivist. As such, meaning and experience was interpreted to be socially produced and reproduced via an interplay of subjective and inter-subjective construction. An experiential orientation to data interpretation was adopted in order to emphasize meaning and meaningfulness as ascribed by participants. A predominantly inductive approach to data analysis was adopted, meaning data were open-coded and participant/data-based meanings were emphasized. Deductive analysis was, however, employed to ensure that open-coding contributed to producing themes that were meaningful to the research questions, and to ensure that the participant/data-based meanings that were emphasized were relevant to the goals of the research. Both semantic and latent coding were utilized in the analysis, with semantic codes produced when meaningful semantic information was interpreted and latent codes produced when meaningful latent information was interpreted. As such, any item of information could be double-coded in accordance with the semantic meaning communicated by the participant and the latent meaning interpreted by the researcher (Patton, 1990).

**Figure 1.**
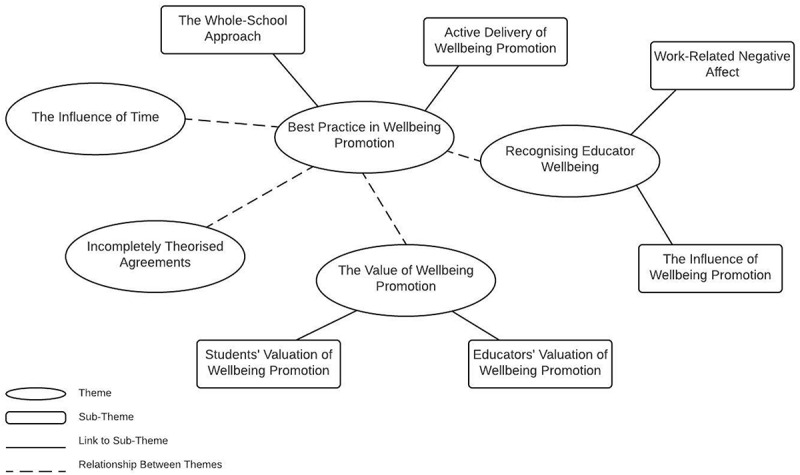
Thematic framework

As an interpretive analytical method, RTA is about “the researcher’s reflective and thoughtful engagement with their data and their reflexive and thoughtful engagement with the analytic process” (Braun & Clarke, 2019, p. 594). Adhering to the interpretive nature of RTA, analysis was primarily conducted by author one. Thus, the results of the analysis represent author one’s interpretations of the data. Author two was employed to audit the reflexive analytical process, sense-check ideas and explore multiple assumptions or interpretations of the data. The aim of this approach was to achieve generalizability of relative concepts as discussed by participants, as opposed to being able to generalize to types of educators (e.g., male, female, teacher, principal etc.) (Creswell, 2009). Analysis was conducted using Microsoft Excel 2016, and adhered to a six phase analytical process (Braun & Clarke, 2020). At phase one, familiarity with the data was pursued by reading all interview transcripts several times, with interviews having been transcribed verbatim. At phase two, initial codes were generated using open-coding. This resulted in a wide array of potential interpretations of the data. At phase three, codes were revised where necessary, and collated under initial themes. A review of these themes at phase four resulted in further iterations of coding, culminating in the interpretation of five themes (Figure 1). Themes were defined and named at phase five, and the report written at phase six.

## Results

### Theme one—best practice in wellbeing promotion

#### Sub-theme one: active delivery of wellbeing promotion

Participants predicated active delivery of wellbeing promotion on the behaviour of educators in identifying and pursuing opportunities to promote student wellbeing and encouraging students to participate in wellbeing-orientated activities. This was said to require that educators demonstrate to their students a willingness and desire to establish close interpersonal relationships, while also attending to their pastoral care role. Participants highlighted numerous casual behaviours that they felt were conducive to establishing referent relationships with students: *“ … it’s about smiling and saying hello to a youngster in the morning. It’s about looking out for that youngster who sits alone at lunchtime” [P3]*. Pursuing relationships with students in this manner informs a greater sense of relatedness among students, and is allied to student motivation and wellbeing (Deci & Ryan, 2000). Participants also believed that this approach was beneficial to their pastoral role. Advocacy for engaging in this type of informal approach was frequently punctuated by the argument that educators need to be “switched on” and looking for potential issues students may not be vocalizing.

*“You can’t just wait for them to come to you with a problem they’re having. You have to be on the lookout for it. You have to talk to them, you know, start the conversation. You have to let them know that you’re there for them” [P11].*

Pro-activity was also advocated with regard to the wellbeing curriculum. Participants argued the merit of students becoming involved in the delivery of lessons as a more relatable and engaging way to deliver the likes of SPHE and CSPE. Participants were aware that the content of these subjects could be quite abstract and might sometimes be difficult for students to relate to and understand. There was also an awareness that students may not understand why some aspects of this curriculum are important. To remedy these issues, participants argued that educators adopt appropriate teaching methodologies to capture and maintain the interest of students. A number of participants endorsed debating as a way to facilitate deeper learning, while also increasing relatability and securing increased student engagement. Drama, or “acting things out”, was also reported to be employed in this regard. Indeed, activities such as debates and drama have been identified as highly appropriate educational pedagogies, and can instil in students a sense of autonomy and ownership of the learning process (Deci & Ryan, 2000).

*“ … act it out (a bullying incident), and then have a silence afterwards. You know, ‘how did that feel? How did that feel to be that person?’ You know, it’s the only way to make it (wellbeing) relatable to students. Some students might be able to take a term and apply it, the vast majority won’t. They need to see it in action” [P4].*

Participants identified a host of other activities they believed were conducive to an active and practical approach to wellbeing promotion. Meditation, mindfulness and yoga were highlighted, as these could be incorporated into SPHE classes. While it was widely appreciated that participation in such activities is beneficial to student wellbeing, the goal for participants was more so to teach these activities so students could use them in the future as needed.

*“ … we definitely need to be teaching them practical ways to look after their own wellbeing. So, things like meditation, mindfulness, body-awareness, I think these are the kind of things we should be teaching. Skills that they can go home and use when needed.” [P1].*

This subscribes to the NCCA (2017, p. 11) dictum that educators should view wellbeing promotion as “a process of ‘well-becoming’, where young people are gaining knowledge, skills, values and attitudes that will sustain them throughout their lives”. In this sense, wellbeing activities are instructional as much as they are remedial. The development of competence in this regard is an important aspect of wellbeing, while the continued investment and engagement in these types of activities has been linked to the actualizing process (Ryff, 2014). More often than not, participants emphasized the importance of this learning over any particular type of activity.

While most participants could easily relate many types of activities to an active process of “well-becoming”, only a small number were also able to relate this process back to the greater curriculum. Some participants recognized woodwork and home economics for their practical nature, which was considered highly conducive to augmenting student wellbeing. Speaking about home economics, one participant said; *“it’s fun and it’s very tactile, and it gets the students working with their hands” [P10]*. An advantageous latent function of these subjects was recognized, in that the delivery of these subjects is inherently active and this activity promotes the development of competence in useful life-long skills. This particular participant portrayed an image of home economics being enjoyable for students and contributing to lifting students’ moods in the moment, while also equipping students with important skills that can be used later in life.

#### Sub-theme two: the whole-school approach

A whole-school approach was proposed to be a fundamental aspect of best practice in wellbeing promotion. The whole-school approach was said to require the involvement of the entire school community, including management, teachers, support staff, and students, but to be driven by a number of key positions including wellbeing coordinators, pastoral care team-members and guidance counsellors: *“you start with—you delegate those ‘official’ roles. Your guidance counsellor, your (wellbeing) coordinator. But, you support them. Everyone gets involved … ” [P7]*. The whole-school approach was said to oblige the entire school staff to have some degree of capability to attend to the wellbeing of their students, with all teaching staff having a working knowledge of relevant policies and curricula. One participant clarified how this requirement was actioned in their school, and how it was beneficial to wellbeing promotion.

*“Our principal wants us all to have some kind of level of … erm … ability … to do the wellbeing thing. Other teachers have done a lot more (training) – the ones that are actually doing the SPHE classes! But, we’ve all been and done something (some training).” [P6].*

The perceived strength of a school’s wellbeing culture and ethos was constructed by participants in relation to the perceived level of staff support, openness (e.g., “an open-door policy”), involvement and leadership, both in relation to students and each other. While factors such as wellbeing activities and educator training were appreciated, culture and ethos seemed to transcend these in terms of importance.

*“ … you know, culture and the way we live that day-to-day, I think is really, really important. And, without that, it doesn’t matter how many hours you put on or say are mandatory … or what you put in a timetable. If that bit doesn’t exist and doesn’t work, you’re wasting your time” [P3].*

Participants often made specific reference to the NCCA (2017) mandate that schools are to allocate a minimum of 300 hours to wellbeing promotion over the course of the junior-cycle. While participants recognized the importance of this mandate, some were quick to suggest the futility of this venture if staff are not inclined towards the promotion of student wellbeing. Indeed, the NCCA (2017) highlight the centrality of school staff in the process of wellbeing promotion, while much research has also highlighted the importance of positive educator perceptions when aiming to deliver positive outcomes regarding student wellbeing (e.g., Forman et al., 2008; Jamtsho, 2015; Schonert-Reichl, 2017).

Participants also demonstrated an acute awareness of the importance of selecting an appropriate educator to deliver the wellbeing curriculum. In addition to being adequately trained, it was proposed that this educator should be enthusiastic about wellbeing promotion, but also be able and willing to engage pro-actively with young people. Moreover, it was considered that this educator should have a personality that would predispose them to the pastoral care inherent in the wellbeing curriculum, and that they should be comfortable with the “difficult conversations” that may invariably arise when discussing sensitive subjects such as substance use, relationships, sex and sexuality. However, concern was expressed that these criteria were often not observed, and that the allocation of an educator to SPHE was sometimes predicated upon availability more so than suitability.

*“So, this is how it works. ‘A P.E. teacher has 24 lessons of P.E. He needs another five lessons. I’ll throw him into SPHE’. Now, that P.E. teacher might be the last person in the world who should be delivering it because, number one, he (hypothetically) doesn’t see any importance in it. Number two, he hasn’t the personality, you know, to deliver the stuff around sex, around drugs and alcohol. People shy away from that. They won’t have hard conversations with youngsters about that, or anything that’s challenging. And that’s the sort of people you should not be letting near SPHE.” [P3].*

Discussions around the topic of choosing an appropriate educator to deliver the wellbeing curriculum tended to be informed by negative connotations. Participants often identified the criteria for what an appropriate SPHE educator is by identifying what an appropriate SPHE educator is not. Particularly at the beginning of discussions regarding this topic, participants were observed to engage in perceived value dissimilarity (Struch & Schwartz, 1989), identifying undesirable criteria and establishing the identity of an appropriate SPHE educator as antithetical to these criteria. Ascribing meaning or value in this way has been strongly associated with negative perceptions of the “other” group (Sirin et al., 2004; Struch & Schwartz, 1989), in this case the inappropriate educator, and could negatively affect a sense of relatedness among colleagues (Deci & Ryan, 2000).

### Theme two—the value of wellbeing promotion

#### Sub-theme one—educators’ valuations of wellbeing promotion

Overall, participants reported an underlying positivity with regard to the promotion of student wellbeing. Participants highlighted the importance they attributed to wellbeing promotion, while also demonstrating a clear appreciation for both short-term and long-term outcomes for students in this regard. In the short-term, there was an awareness of the immediate impact the school environment can have upon students’ wellbeing. This is widely documented in the literature, with social and academic stressors being particularly salient in the post-primary context in Ireland (O’Brien 2008; Smyth 2017). In the long-term, the value of wellbeing promotion was attributed to the preparation of students for their adult lives, which is also supported by previous research (Seligman et al., 2009; Vaillant & Davis, 2000).

*“Well, I fully agree with making it mandatory! I think it’s such an important part of their learning, and their school-day. I think – I’ve believed for a very long time that we need to be doing more to prepare them – to prepare students for this aspect of … of life!” [P9].*

Participants presenting with an underlying positivity regarding wellbeing promotion is in line with previous international research (see Apostolidou & Fontana, 2003; Doyle, 2017; Maloney et al., 2016). However, a number of concerns were voiced regarding a perceived systematic de-prioritization of the wellbeing curriculum. One participant drew on their experience of working with a colleague who was assigned to deliver SPHE, but did not afford much value to the wellbeing curriculum.

*“ … like I’ve said, students can come in ready for their doss class. But, equally, if you get the wrong teacher doing it (delivering SPHE), they could be thinking the same! They might be thinking, ‘I can catch up on my paperwork here’” [P5].*

Over the course of this discussion, participant five mused that their colleague’s lack of sufficient value for SPHE resulted in this colleague abstaining from delivering this class in an appropriate manner. Further, it was suggested that this colleague attended to other responsibilities during this class. Educators who under-value wellbeing promotion have been found to be resentful of losing academic activities in favour of wellbeing promotion, viewing the wellbeing curriculum as encroaching upon the core curriculum. Indeed, previous research has found that, even when educators valued the wellbeing curriculum, they often afforded it “low status” relative to the core curriculum (Mayock et al. 2007; Doyle, 2017). This trend was observed among participants in the present study. The value of the wellbeing curriculum was said to be easily recognizable, but delivering the core curriculum was considered a more pressing priority: *“I suppose you could say that, you know, you’re trying to teach the content of the course and get them through exams, and that’s your priority. Then, wellbeing isn’t the priority” [P4]*. This participant—an SPHE teacher—offered that the core curriculum might be afforded more legitimacy due to the associated measure of formal assessment.

While an underlying positivity regarding wellbeing promotion and related curricula was noted among participants, the same could not be said for the wellbeing guidelines. Participants viewed the wellbeing guidelines with a degree of scepticism, frequently identifying perceived limitations in terms of the scope of the guidelines’ potential application. The value of the guidelines was considered to be relatively short-term, and their use erstwhile. Participants often referred to the wellbeing guidelines in the past tense, indicating that they and their school had “moved on” from the instruction the guidelines provide.

*“I mean, they don’t really have longevity. We brought our school in line with the guidelines and that was kind of it. You know, we haven’t really – we don’t go back to them that often.” [P11].*

While participants bemoaned the lack of longevity of the wellbeing guidelines, there were numerous accounts of how the guidelines were used to “audit” wellbeing practices. Some participants indicated that this audit was a one-time affair: *“ … we sat down with them (the guidelines) and we checked, you know, ‘what are we not doing? What is this telling us is important that we haven’t covered?’ and we made some adjustments” [P11]*. However, others said their school would turn to the guidelines periodically as a form of self-assessment: *“ … it might be useful to go back periodically to check that you haven’t slipped, you know, that you’re still doing what you’re supposed to be doing” [P8]*. A tendency to under-value wellbeing- or SEL-related practices can typically result from a lack of knowledge and understanding as to how such practices can create a healthier learning and working environment within schools (see Byrne et al., 2018; Mayock et al., 2007; O’Higgins et al., 2013). If indeed participants’ schools audited their wellbeing practices using the guidelines, the longevity of the guidelines would arguably be apparent in the subsequent reforms to the schools’ wellbeing policies and practices. In this sense, it may be that participants have not recognized potential improvements in their schools, or have not attributed improvements in wellbeing policies and practices to the audit, which was informed by the guidelines.

#### Sub-theme two—students’ valuations of wellbeing promotion

There was a perception among some participants that students very often did not value wellbeing promotion or related curricula. Such participants frequently conjectured that students *“don’t really see the point in all this wellbeing stuff” [P8]*, while also postulating a predominant perception among students that SPHE did not require much effort and was “a bit of a doss class”. These participants identified an intrinsic devaluing of wellbeing promotion among students, proposing that students simply did not see the value in such activities. Conversely, other participants attributed a perceived under-valuing of SPHE among students to oversaturation of wellbeing education across the greater curriculum. Cited examples were puberty and substance misuse being discussed physiologically in both science class and SPHE. Students’ negative perceptions of SPHE were then attributed to duplication across the greater curriculum, more so than an intrinsic de-valuing of SPHE.

*“You know, wherever there’s something in the SPHE class that’s in one of the other subjects, I’d take it out of the SPHE class. Or, at least re-write it – re-do it so it has more of an emphasis on how it relates to wellbeing” [P6].*

The prevailing degree of negativity participants perceived among students is not wholly reflected in the available literature. Research in this area has tended to find mixed perceptions among students in this regard. For example, in a case study conducted across 12 schools, Nic Gabhainn et al. (2010) documented a negative sentiment among many students, with some referring to SPHE as boring, useless and unhelpful. However half of the students considered SPHE to be an important part of what they learned, with 41% considering SPHE to be as important as core curriculum subjects. Participants in the present study frequently proposed that many aspects of the wellbeing curriculum might not be relatable for students and that students might have difficulty seeing the value in these types of lesson.

*“ … it can be difficult for some pupils to relate it (the wellbeing curriculum) to their wellbeing – to see how some of the topics relate to their wellbeing. So you have to find ways to make it relate … you know, make it relatable” [P8].*

Many participants argued that the wellbeing curriculum needed to be updated to remove duplication and to be more practical and relatable for students. Indeed, a more relatable curriculum delivered using engaging pedagogies would arguably facilitate students’ sense of relatedness with regard to the subject matter, as well as their sense of autonomy over the learning process (Deci & Ryan, 2000; Mannix McNamara et al., 2012).

Similar concerns were also raised about the wellbeing guidelines. Particularly concerted critiques were levied against the indicators of wellbeing, in that they were considered unrepresentative of how young people perceive, discuss, or attend to their wellbeing.

*“I don’t know if you want me to talk about the wellbeing indicators [interviewer nods]. I have them there on my wall – this is maybe my third year to have them on the wall. To be honest, I feel that that’s just way too abstract! It means nothing to a 13 or 14 year old, absolutely nothing” [P4].*

For participants, the inability of students to relate to the wellbeing guidelines was an important factor in their respective valuation of wellbeing promotion. Indeed, student involvement in SPHE can be a correlate of augmented student value-perceptions, and has been widely advocated across the literature (see Mayock et al., 2007; Moynihan & Mannix McNamara, 2012; O’Higgins et al., 2013). Such studies have highlighted the benefits of including the views and experiences of students in developing, reviewing and delivering SPHE and wider SEL policies, practices and curricula. In the present study, participants intimated a perception that student involvement in wellbeing promotion was largely absent, and that this may have contributed to students’ de-valuing wellbeing promotion.

### Theme three—the influence of time

When discussing time constraints in relation to wellbeing promotion, the first port of call for many participants was to highlight their workload. Participants reported that their workload had been increasing over the years, often in very small increments: *“So, you know, little by little, there’s lots of work being added on … every teacher is going to say that” [P4*]. Some participants reported sometimes working significant amounts of overtime due to consistent increases in their workload. Participants reported that this issue seemed to have been compounded by the formalization of wellbeing promotion, with some proposing that wellbeing promotion has significantly increased their workload.

*“I mean, the workload has increased since wellbeing was introduced. You know, there’s so much … administration. There’s a lot of meetings, and meetings with parents. And, we’re doing that on top of everything we were doing. Nothing has been cut back to make room for it” [P8].*

The majority of the additional workload reported seems to be in relation to meetings and administration, as opposed to wellbeing-oriented activities. This was further conveyed by other participants, who reported an increase in workload, but little perceptible change in terms of wellbeing-orientated activities: *“We’re pretty much doing the same thing (to promote wellbeing), but there’s the extra hours that have to be included. And there’s a lot of reporting!” [P10]*. This participant spoke of workload extensively over the course of their interview and concluded that workload was the single most inhibiting factor with regard to the use of appropriate pedagogic practices. This participant also felt that the way in which they were charged with promoting student wellbeing was burdensome and uninformed, and further contributed to an unsustainably heavy workload. When asked how the issue of educator workload could be addressed, participant ten replied, “*Just reduce it! Stop adding to it! Don’t just extend the curriculum without understanding the impact on our ability to teach the curriculum!” [P10]*. The sentiment communicated here was that workload has increased with little consideration for the impact upon educators’ ability to actually do the work. When workload becomes overburdened in this way, SEL activities typically become de-prioritized in favour of academic activities, and are subsequently afforded less time in the school day (Barry et al., 2017).

Participants reported that heavy workload could sometimes result in extra hours of work. Frustration and dissatisfaction was evident in relation to educators undertaking work-related tasks during lunchtime, or when such tasks were undertaken outside of the school context: *“work does come home with you! I’m forever marking tests at home because there’s just no other time to do it!” [P10]*. Inconsistencies were also reported in the way measures that were introduced in order to improve student wellbeing inadvertently contributed to heavy workload as an inhibitory factor in relation to wellbeing promotion: *“ … ironically, with this wellbeing stuff, we’ve more forms to fill out, so we’ve even less time (for wellbeing promotion) now than we did before!” [P5]*. The increased wellbeing-related meetings, reports and administrative tasks were seen to be particularly burdensome. Brady and Wilson (2020) proposed that such heavy monitoring and accountability with regard to educators’ performance and activities may not only act as a barrier to student wellbeing, but can also be detrimental to educator wellbeing, as they may feel they are working to provide evidence of their competence rather than achieving optimum outcomes for their pupils. Conversely, participants rarely communicated negative perceptions of affording additional hours to students’ wellbeing. While there were no examples offered in terms of attending to student wellbeing outside of the school context, there were many examples of using lunchbreaks in this regard. At a minimum, participants spoke of giving up their lunchbreak in a perfunctory, matter-of-fact manner: *“some kids don’t have friends! So then, you’re their friend. You’ll try to keep them company during lunch” [P6]*. Others found sharing their lunch with students to be an enjoyable experience.

*“I’d often spend my lunchbreak – part of my lunchbreak in the lunchroom. You know, I’d bring my cup of tea in and just have a chat with the pupils that are there. I think – I mean I love it! I have really good fun with them! But, I think it really builds that trust, you know? It lets them know that you’re there to talk” [P11].*

This participant described how educators taking lunch with students could be reciprocally beneficial for both parties. While a trusting referent bond may be established with students, the participant also professed deriving pleasure from socializing with students. Participant eleven seemed to lean into the pursuit of “dual relationships”, whereby the participant attended to the needs of their students, while also soliciting validation with regard to their professional or personal self-concept (Davis, 2006). While these types of relationships have been found to be an important source of enjoyment and fulfilment in educators’ careers (Hargreaves, 2000; Spilt et al., 2011), attending to student wellbeing in this manner is nonetheless a further undertaking of emotional labour. Although this participant spoke with positivity and optimism, for some educators, the lack of reprieve from emotional labour could hasten the onset of burnout, to which educators have been found to be acutely susceptible (Kinman et al., 2011).

It was also widely reported that attending wellbeing-related CPD could be difficult because of time constraints resulting from high workload. This was seen to be particularly problematic for non-wellbeing educators: *“I’m not an SPHE teacher so I don’t really get to go to those training workshops. I wouldn’t really have time anyway!” [P2]*. The apparent solution to CPD-inhibiting time constraints seems to have been to provide CPD courses and workshops outside of school hours. However, non-wellbeing educators may be reticent to undertake formal CPD outside of what they viewed to be their contracted hours.

*“I know there are CP – eh, continuous professional development programmes but, again, where can you fit them in. We shouldn’t really be expected to do CPD in our own time” [P1].*

Due to an inability to free up time to be available for wellbeing CPD during school hours, non-wellbeing educators might often only be able to avail of courses and workshops that run outside of their contracted hours. Considering their explicated reticence to afford personal time to wellbeing-related CPD, non-wellbeing educators may be somewhat under-prepared for the task of attending to student wellbeing.

### Theme four—incompletely theorized agreements

It was evident that all participants were aware of the existence of the NCCA wellbeing guidelines and that they believed their colleagues were also aware of the guidelines. However, participants were often very forthcoming in admitting that they or their colleagues have likely not engaged with the guidelines to a sufficient degree: *“I think every teacher is aware of it. They wouldn’t have read the guidelines, but I think we’re all aware that there are wellbeing guidelines there to be read if anybody wants to read them” [P4]*. This participant spoke of engagement with the wellbeing guidelines as if it were an optional exercise at the discretion of the educator. While participants frequently spoke of time constraints inhibiting wellbeing promotion in numerous ways as per theme three, it seems that a lack of perceivable value for the wellbeing guidelines may have informed participants’ non-engagement with the wellbeing guidelines, as per theme two. As such, not only were participants often unfamiliar with the wellbeing guidelines, but they were also unsure as to how the guidelines may be utilized in their school.

*“Well, I presume I’m implementing the guidelines. You know what I’m going to do now. I’m going to go away and read them [laughs]. Erm [long pause], look, whatever we’re supposed to do from a mandatory point of view, I know we’re doing, right! That’s being covered. But, I would like to think, as a caring school that cares about its youngsters, that whatever the guidelines are, we’re doing that and more! And I’m hoping that when I read them I go, ‘yeah, yeah, yeah’ (implying all areas of the guidelines are covered)” [P3].*

Participant three assumed that, “as a caring school”, what the staff are doing to attend to student wellbeing naturally overlaps with mandated policies and practices. However, the participant was evidently uncertain that this is indeed the case. It is telling that discussions with this participant (and others) regarding student wellbeing were largely atheoretical over the course of the entire interview. For example, there were numerous references to educators “putting their mammy-head on”, but few discussions of appropriate policies or formal practices. To some degree, this participant’s school seems to have adopted a “generally accepted body of values” in relation to the promotion of student wellbeing. This term was first used in 1950’s post-war America to describe an informal set of moral values that were to be disseminated through the country's school system in order to inculcate in young people a sense of American identity and citizenship (McClellen, 1992). In the context of participant three’s school, this would be a micro-functionality of the school staff informally agreeing upon a wellbeing value system in the absence of consideration for, or awareness of, appropriate theory.

Perhaps unsurprisingly considering their difficulties in availing of wellbeing-related CPD and their concerns regarding their lack of knowledge of wellbeing, participants felt their level of wellbeing-related training was inadequate and needed to improve. There was a clear sense that, considering the now mandatory nature of wellbeing promotion, the way in which wellbeing-related CPD is provided needs to be improved and that such training should be available to all staff members: *“I think ‘being tasked’ with it (promoting student wellbeing) changes things a bit. I think there needs to be a bit more support across the board. There needs to be more training for everyone” [P9]*. One participant argued the necessity of a corresponding mandate for training, particularly among wellbeing educators: *“make it (CPD) mandatory for anyone who will be delivering wellbeing lessons … ” [P1]*. The position that wellbeing educators required more training than did non-wellbeing educators was largely shared among participants. Non-wellbeing educators did recognize that wellbeing educators were more acutely responsible or accountable for SEL aspects of wellbeing promotion and recognized the corresponding requirement for additional, more specified, training. Non-wellbeing educators seemed to be content with the idea of receiving more generalized training that would facilitate their day-to-day function as educators who informally monitor student wellbeing: *“ … for the rest of us (non-wellbeing educators), maybe it’s not so much about the (wellbeing) curriculum. Maybe it’s more about the day-to-day stuff.” [P2]*. Compelling arguments were also made against such heavy dependence upon CPD to inculcate in educators an appropriate level of expertise to be sufficiently capable of attending to student wellbeing.

*“CPD is all well and good, but it actually shouldn’t be necessary, you know – or as necessary! Teachers should be able to confidently and competently teach SPHE from the start of their career. They shouldn’t have to upskill on the job, you know?” [P9].*

This participant—an SPHE teacher—presented a compelling summative statement of the current state-of-the-art in terms of SPHE training. Educators appear to be required to begin developing a capability to deliver SPHE subsequent to the commencement of their career. The implication of this statement is that insufficient training in SPHE is available for pre-service educators enrolled in teacher-educator programmes. This is reflective of previous research, which highlighted that while some third-level institutes offer postgraduate courses that address SPHE to some degree, most do not. Further, variation in the provision of SPHE training was noted at an undergraduate level, with some institutes offering little or no exposure to such training. It was strongly recommended that health education receive more consideration on the curriculum for pre-service teachers (Mannix McNamara et al., 2012). Considering the proposition made by participant nine, it can be considered that as of yet, this issue remains unresolved. As such, when pursuing wellbeing-related practices, educators would seem to be often reliant upon what Sunstein (1995) termed “incompletely theorized agreements”, which posits the way in which individuals or groups can work together to pursue an action in the absence of theoretically informed agreements as to why that action may be appropriate.

### Theme five—recognizing educator wellbeing

#### Sub-theme one—work-related negative affect

One of the most prominent contributory factors to participants’ experience of work-related negative affect was the stress and difficulty of managing the classroom, and addressing students’ behavioural issues. When asked which aspect of their day-to-day responsibilities they found to be difficult or stressful, one participant replied *“ … probably controlling the class. When someone is acting up in any class […] you have to put a stop to that pretty quick” [P2]*. Classroom management that tends towards the immediate correction of undesirable behaviour cedes from pedagogic merit or instruction that may be beneficial to student learning outcomes (Eisenman et al., 2015). Such directly controlling teacher behaviours (DCTB’s) can not only negatively affect student autonomy, but can also be harmful to student/teacher relationships (Pikó & Pinczés, 2015). Several participants used somewhat authoritarian language when discussing classroom management. In particular, there were several references to “keeping on top of” behavioural issues, which implies an adversarial view in terms of classroom management.

*“Keeping on top of the class is a nightmare sometimes! […] and that can get to you. I’m at the stage now where I wouldn’t let it make me doubt my ability to deliver my subjects. But, it doesn’t mean it doesn’t stress you out a bit” [P5].*

The way in which participants tended to speak of classroom management being “a nightmare” and “very difficult” suggests a susceptibility to engaging in DCTB’s in order to bring a swift end to adverse conditions. Participant five identified that attending to disruptive behaviour can be a source of stress, but suggested that their level of experience as an educator helped to insulate against a potential threat to their self-concept as a teacher. Indeed, Eisenman et al. (2015) drew upon numerous studies in highlighting that early-career teachers believed weak classroom management skills to be among the most significant threats to their self-concept as a ’good teacher’. Participants viewed classroom management as a significant requisite skill for all educators, and considered the absence of such skill to be contributory to the onset of increased stress. To this end, several participants proposed that increased support with regard to classroom management would have a positive impact upon educator wellbeing: *“I think maybe more supports for teachers in terms of discipline. A lot of teachers find the discipline a big stressor in their lives” [P4].*

Another pressing concern was the difficulty often experienced by educators when they were required to interact with parents. Many participants reported sometimes finding it very difficult to secure parental involvement and establish an amicable relationship within which to work through a given issue. This resonated among several participants. One participant captured the trepidation with which educators sometimes made the initial call to a pupil’s home.

*“Parents!! Gosh, don’t start me! Parents are like a double-edged sword. They can be either – you can get these parents who are absolutely brilliant! And, you can lift the phone to them and talk to them about anything about their child. And there’s these (parents) then that you would nearly talk to a lawyer before you would lift the phone to them. Some parents are really difficult to deal with!” [P3].*

Participants were cognizant of the importance of working amicably with parents in order to achieve optimum outcomes for their students. Research conducted in elementary schools in the USA argued beyond the need for amicable relationships, stating the importance of congruence in teacher and parent perceptions of appropriate academic, social and behavioural outcomes for students (Minke et al., 2014). Historically though, educators have been found to interpret a perceived lack of parental involvement in the school context as an absence of parental support for their children’s education, which was said to precipitate unproductive parent/educator working relationships (Lawson, 2003). This phenomenon was also observed among participants, with some reporting sometimes feeling censured by parents.

*“ … first; it’s nearly impossible to get them down to the school to talk to them, second; you just get an earful! They don’t want to hear it! It’s always your fault! It’s always your responsibility! It’s your job, and you’re not doing it properly! But, it’s their job! Ultimately, it’s the parents’ job to raise their kids!” [P6].*

Participant six communicated a perception that some parents hold educators entirely accountable for student outcomes. However, this participant also demonstrated a propensity towards recrimination by highlighting that “ultimately, it’s the parents' job to raise their kids„. Drawing from several international studies, Miller (2003) proposed that prominent factors inherent in difficult parent/educator relationships were mutual suspicion, recrimination and blame. Miller found that both parents and educators tended to be suspicious of the other party’s level of involvement, while also attempting to attribute blame to the other party for shortcomings or misgivings in achieving positive outcomes for students. There was much evidence in the data of the present study that relationships between parents and educators could often be tense and sometimes adversarial. Miller (2003) argued that these types of relationships are not uncommon and can very often stem from communication difficulties. Miller further argued that communication difficulties could be exacerbated when educators lack certain interaction skills. This was also evident in the present study, with some participants suggesting that upskilling in this area would be advantageous when working with parents.

*“ … maybe if there was some support or guidance on how to deal with difficult parents […] Or a workshop on how to deal with those parents” [P10].*

Most participants tended to advocate increased training and support as the most appropriate measure to improve educator wellbeing: “*The way to help my wellbeing in work is to address the sources of stress … give us some support” [P2]*. The degree to which educators appear to be over-stressed and under-supported with regard to their wellbeing has, by one participant’s account, led to a widespread tendency towards school refusal among educators: *“ … a lot of teachers do feel very stressed. Some schools, you’ll find there’s huge absenteeism among teachers” [P4]*. This is consistent with previous research, which identified occupational stress, as well as job dissatisfaction, to be strong predictors of absenteeism among Irish post-primary educators (Ennis, 2019). Moreover, research conducted in England found that teachers are more likely to report symptoms of stress and depression than are the general population (Health and Safety Executive 2017), and that such teachers were twice as likely to have taken sick leave in the preceding month compared to colleagues with fewer wellbeing concerns (Kidger et al., 2016). More strikingly, it has been suggested that poor wellbeing is the primary contributory factor in teachers’ decisions to leave the profession (CooperGibson, 2018).

#### Sub-theme two—the impact of wellbeing promotion

Participants were able to identify several ways in which the requirement to attend to student wellbeing could directly bear a negative influence upon their own wellbeing. Principal among these was the effect of perceived inadequacies in training regarding wellbeing promotion upon participants’ sense of job satisfaction. This was communicated as a lack of confidence that actions pursued by participants to address a given wellbeing concern were, in fact, beneficial in terms of the student’s wellbeing.

*“You know you can draw on your experience as a teacher, and you know, even if you’re a parent, that can help. But, especially now that it’s (wellbeing promotion) mandatory, you could do with having that bit of security that you’re doing the right thing. You know, not having any kind of training or background information can give you that little bit of a doubt that maybe you’re not handling that situation so well” [P9].*

Participant nine implicated a lack of appropriate training and requisite knowledge in their insecurity with regard to wellbeing promotion. To this end, participant nine discussed calling upon any relevant experience that may be of use, which in this example was their experience as a teacher and a parent. This is reflective of Sunstein’s (1995) thesis of incompletely theorized agreements, as participant nine acknowledged the absence of what Sunstein (1995, pp. 1740-1741) might refer to as “high-level theory” of wellbeing, and subsequently resorted to “low-level principles” of good caring. This seemed to bring participant nine to an acute awareness of a lack of preparedness to attend to the task of promoting student wellbeing. This phenomenon was observed among several participants. When questioned further as to how this lack of preparedness made them feel, participants shared concerns regarding their self-image as a “good teacher”.

*“ … doing the SPHE, I know I’m not doing a good job at it! It’s just – it needs to be taught differently and I don’t know – I never learned how to do that. It doesn’t feel good to not be good at your job. It can knock your confidence” [P2].*

Accounts of participants’ feelings regarding a lack of preparedness to deliver wellbeing promotion suggest a threat to their ability to develop a sense of competence in this regard (Deci & Ryan, 2000). The implications of an inability to achieve a sense of competence was not lost on participants, with some indicating an understanding of potential negative outcomes for both students and educators: *“ … it’s bad because, not only does it affect the students and their ability to learn this stuff, but … it kind of knocks your confidence!” [P2]*. Participant two’s observation is very much in line with the student/teacher dialectical framework, as educators’ negative perceptions of their levels of competence may not only be deleterious to their own self-image, but can also precipitate the abandonment of teaching methods and pedagogies that students find most engaging (Deci & Ryan, 2000).

Participants argued that many of their concerns could be addressed with appropriate training. Participants were also aware of the reflexive benefits appropriate training could have in terms of their own wellbeing, particularly with regard to reducing stress and promote a healthy self-image: *“The wellbeing thing is causing stress because I’m not trained, and I don’t feel confident with it. If I was trained, it wouldn’t be so stressful, and that would then benefit the students because I could do a better job delivering the lessons!” [P1]*. However, some participants expressed a degree of hopelessness and resignation in terms of their ability to overcome many of the barriers to best practice in promoting student wellbeing. For example, one participant spoke of their acquiescence to the reality that they were simply unable to secure the investment of some students: *“Some pupils just don’t want your help. And, there’s just no getting through to them. […] You get to a point and you just have to be like; ‘ok, I tried’” [P8]*. Another participant presented with an external locus of control with regard to educators’ ability to avail of appropriate training: *“I mean it’s kind of out of my hands. There are courses that come up, but it’s nearly impossible to make time to go to them. I don’t think I can do much to fix this issue … ” [P1]*. The inability of some participants to identify ways to overcome these obstacles potentially presents the reality that participants are, to some degree, incapable of bringing about desired outcomes and that they may not feel satisfied in the ownership of their actions. This would represent a significant threat to participants’ sense of competence and autonomy (Deci & Ryan, 2000), which would be a reflexively compounding barrier to achieving optimum states of wellbeing for both students and educators.

## Discussion

Analysis of the data in this study indicated that, overall, post-primary educators seem to view the promotion of student wellbeing as a valuable and worthwhile task. Conversely, attitudes regarding the NCCA wellbeing guidelines tended to be somewhat negative, if not uninformed. Participants argued that the wellbeing curriculum needed to be updated and more relatable for students. A lack of appropriate training, work-related time constraints and the de-valuing of the wellbeing curriculum relative to the core curriculum presented as the most salient barriers to the achievement of best practice in wellbeing promotion. These factors also appear to threaten educator wellbeing. The success of the measures in place regarding student wellbeing is greatly contingent upon an appropriate knowledge and skillset, as well as positive attitudes and perceptions, among educators (see Byrne et al., 2018; Mayock et al., 2007; NCCA, 2017). It is instructive to consider that participants advocated increased training and reduced workload as the most suitable measures to improve their wellbeing. In this regard, the argument can be made that the most appropriate action to address educator wellbeing, augment educators’ attitudes and perceptions of wellbeing promotion, and ultimately improve the promotion of student wellbeing, would be to facilitate educators in redressing shortcomings in training, time constraints and value regarding the promotion of student wellbeing.

## Practical implications

Although wellbeing has been a recognized area of learning in Ireland since 2015, the wellbeing curriculum occupies an unusual grey area among relevant stakeholders. For example, the 2017 draft of the teaching council registration “curriculum subject requirements”, which outline the requisite skills and accreditation to deliver each subject of the post-primary curriculum, only addresses two (CSPE and PE) of the three subjects that comprise the wellbeing curriculum (The Teaching Council, 2017). As of yet, SPHE is not recognized. It is therefore recommended that appropriate subject requirements be established for SPHE, which prospective teachers would be required to meet in order to register with the teaching council to deliver this subject (see Appendix B). This would oblige all consecutive and concurrent teacher-education programmes to offer a learning pathway that would lead to an accredited qualification in teaching SPHE.

As wellbeing promotion is conceptualized as a whole-school practice, for which all educators are responsible (NCCA, 2017), it is arguable that all educators should be provided with the necessary knowledge and skillset to attend to this task. It is therefore also recommended that a requisite base-level wellbeing-orientated knowledge and skillset be mandatory for all educators in order to register as a post-primary teacher with the teaching council, regardless of their chosen subject. This would require that all prospective educators undergo some degree of pre-service training and that this training be recognized as a requisite criterion to register as a teacher with the teaching council. For example, this training could take the form of a mandatory “wellbeing” module(s) on all accredited concurrent and consecutive teacher-education programmes.

The benefits of the recommended measures would potentially be wide reaching, accounting for best practice and both student and educator wellbeing. The introduction of an accredited SPHE learning pathway would help to ensure theoretically informed best practice in delivery of the wellbeing curriculum. Such a programme would reduce reliance on CPD and circumnavigate time constraints as a barrier to adequate training by establishing an appropriate level of knowledge and skill pre-service. Accreditation requirements would potentially reduce the propensity for “inappropriate educators” to deliver SPHE, and would insulate educators from being “thrown into” SPHE to fill free hours. A requisite base-level of pre-service training would help to ensure theoretically informed best practice with regard to the implementation of whole-school approaches to wellbeing promotion, and could facilitate a more appropriate wellbeing culture and ethos in schools. Such training could also augment educators’ attitudes and perceptions regarding the wellbeing curriculum in relation to the core curriculum, and help to redress the imbalance of value afforded to each of these curricula.

## Limitations and further research

While the sample in this study was demographically diverse, the sample size was relatively small. Lockdown measures enacted during the Covid-19 pandemic of 2020 curtailed the possibility of further recruitment of participants. The option to conduct a second round of recruitment for online or telephone interviews was not pursued, as feedback from the two participants who did avail of this option, and the participant who withdrew from the study, highlighted severe time constraints in relation to working from home. It should also be noted that all-boys schools were not represented in the sample. In terms of educator position, the sample was also largely homogenous and over-representative of teachers. Nevertheless, there was a relatively diverse sample in terms of the gender, location, subjects taught and wellbeing position of participants. Further, participants offered rich, in-depth accounts of their attitudes and perceptions regarding a range of factors implicit in the promotion of student wellbeing. Furthermore, the present study valued conceptual rather than numerical generalizability. Considering the degree of congruence noted between the findings of this study and the findings of noted previous research, it is apparent that conceptual generalizability was achieved (Creswell, 2009). Finally, it should be noted that one interview was conducted by telephone, and that non-verbal communication could not be recorded in this interview.

As the present study adopted an experiential approach to data analysis, useful future research might involve a critical perspective to examine the socio-cultural factors that underlie the meaning ascribed to wellbeing promotion by educators, and examine the meaning-making process. Alternative research methods might also be employed to build upon the findings of this study. Ethnographic methods could be adopted to observe the lived experience of educators with regard to both the implementation of whole-school wellbeing practices and the delivery of the wellbeing curriculum.

## Conclusion

As outlined in the introduction to this paper, the success of measures aimed at promoting student health and wellbeing is greatly informed by the attitudes and perceptions of the educators assigned to implement these measures. The aim of this study was to conduct a holistic examination of the attitudes and perceptions of Irish post-primary educators with regard to the promotion of students’ social and emotional wellbeing. While the sample size was small, rich data was gathered regarding a broad range of factors relating to the promotion of student wellbeing. This data shows good continuity with previous national and international research. The recommendations of this study are also consistent with several previous national studies in calling for increased pre-services training in health and wellbeing promotion. This study makes a novel contribution to this body of research by advocating for the mandatory nature of such training for all pre-service educators and recommending the development of an appropriate pre-service learning pathway that would lead to an accredited qualification to teach SPHE at post-primary level.

## Appendix A Interview Agenda

Wellbeing Promotion

1. What are your thoughts regarding the inclusion of wellbeing promotion in the junior-cycle curriculum?
2. What do you believe is the best way to promote student wellbeing? *>Elaboration—What is necessary to ensure student wellbeing is properly attended to? What should the WB curriculum be teaching? How should it be taught? What do you need for you to be able to deliver wellbeing promotion?<*
3. Have you encountered any issues that have made it more difficult for you to attend to the wellbeing of your students? What are these issues?
4. Are there any external factors, in terms of your students’ wellbeing, that you feel may be influential in the school setting?

Wellbeing curriculum

1. How do you feel about the current wellbeing curriculum?
2. What training or instruction did you receive in terms of delivering the wellbeing curriculum?
3. Are there any challenges or difficulties that you have encountered in delivering the wellbeing curriculum? What are these challenges? Did you overcome this challenge? How?
4. Are there any changes that you would recommend that you feel might improve the delivery of the wellbeing curriculum? What about wellbeing curriculum itself?

Wellbeing Guidelines

1. How did you become aware of the NCCA wellbeing guidelines?
2. What training or instruction did you receive in terms of how to use the information contained in the wellbeing guidelines to promote student wellbeing?
3. In terms of promoting student wellbeing, how are the guidelines utilized in your school?
4. Are there any changes that you would recommend that you feel might improve the implementation of the wellbeing guidelines? Are there any changes that might improve the wellbeing guidelines themselves?

Educator Wellbeing

1. How do you feel about being tasked with promoting student wellbeing?
2. What are some of the difficulties that you, as an educator, would face while attending to the day-to-day wellbeing of your students?
3. In light of the recent move towards attending to student wellbeing, do you feel that your wellbeing is adequately attended to?
4. How would you like to see your wellbeing accounted for?

I have asked all of my questions. Is there anything you would like to add regarding the topics we have discussed today?

## Appendix B—Draft SPHE Curricular Subject Requirements

*(Note: The criteria set forth for an accredited SPHE learning pathway are drafted based on existing curricular subject requirements. Topics of study listed in section two are hypothesized based on available research (including the present study) and areas of learning in SPHE classes).*

In order to meet the registration requirements set down in the Teaching Council [Registration] Regulations in respect of the curricular subject of SPHE, an applicant must meet **all** of the following criteria:

1. (a) Applicants must hold a degree-level qualification, with SPHE studied up to and including third-year level or higher (or modular equivalent).

(b) The qualifying degree must be equivalent to at least Level 8 on the National Framework of Qualifications (NFQ) and with a minimum pass^1^ result in all examinations pertinent to the subject of SPHE.

(c) The qualifying degree must carry at least 180 ECTS (European Credit Transfer System) credits (or equivalent) with the specific study of SPHE comprising at least 60 ECTS credits (or equivalent) and with not less than 10 ECTS credits (or equivalent) studied at third-year level or higher (or modular equivalent).

1. The study of SPHE during the degree must show that the holder has acquired sufficient knowledge, skills and understanding to teach the SPHE syllabus^2^ to the highest level in post-primary education (see www.curriculumonline.ie). To meet the requirement the degree should have 60 ECTS credits in SPHE, to be comprised by the study of an appropriate selection of the following topics:

**Theory**

• Psychology of Development

• Psychology of Education

• Sociology of Development

• Sociology of Education

• Theories of Wellbeing

• Emotional Intelligence

**Methodology**

• Introduction to Wellbeing Pedagogies

• Advanced Wellbeing Pedagogies

• Wellbeing-orientated activities

• A Whole-School Approach to Wellbeing

• Self-care

**SPHE Modules**

• Gender, Sex and Sexuality

• Wellbeing and Technology

• Substance use and personal safety

• Friendships and Identity

1. Applicants must also have completed a programme of post-primary initial teacher education (age range 12–18 years) carrying a minimum of 120 ECTS credits (or equivalent). The programme should include a methodology module(s) on the teaching of SPHE carrying a minimum of 5 ECTS credits (or equivalent).^3^
